# First-line antiretroviral drug discontinuations in children

**DOI:** 10.1371/journal.pone.0169762

**Published:** 2017-02-13

**Authors:** Melony Fortuin-de Smidt, Reneé de Waal, Karen Cohen, Karl-Günter Technau, Kathryn Stinson, Gary Maartens, Andrew Boulle, Ehimario U. Igumbor, Mary-Ann Davies

**Affiliations:** 1 Division of Clinical Pharmacology, Department of Medicine, University of Cape Town, Cape Town, South Africa; 2 Centre for Infectious Disease Epidemiology and Research, School of Public Health and Family Medicine, University of Cape Town, Cape Town, South Africa; 3 Empilweni Services and Research Unit, Department of Paediatrics and Child Health, University of the Witwatersrand, Johannesburg, South Africa; 4 Médecins Sans Frontières, Khayelitsha, South Africa; 5 US Centers for Disease Control and Prevention, Pretoria, South Africa; Laurentian, CANADA

## Abstract

**Introduction:**

There are a limited number of paediatric antiretroviral drug options. Characterising the long term safety and durability of different antiretrovirals in children is important to optimise management of HIV infected children and to determine the estimated need for alternative drugs in paediatric regimens. We describe first-line antiretroviral therapy (ART) durability and reasons for discontinuations in children at two South African ART programmes, where lopinavir/ritonavir has been recommended for children <3 years old since 2004, and abacavir replaced stavudine as the preferred nucleoside reverse transcriptase inhibitor in 2010.

**Methods:**

We included children (<16 years at ART initiation) who initiated ≥3 antiretrovirals between 2004–2014 with ≥1 follow-up visit on ART. We estimated the incidence of first antiretroviral discontinuation using Kaplan-Meier analysis. We determined the reasons for antiretroviral discontinuations using competing risks analysis. We used Cox regression to identify factors associated with treatment-limiting toxicity.

**Results:**

We included 3579 children with median follow-up duration of 41 months (IQR 14–72). At ART initiation, median age was 44 months (IQR 13–89) and median CD4 percent was 15% (IQR 9–21%). At three and five years on ART, 72% and 26% of children respectively remained on their initial regimen. By five years on ART, the most common reasons for discontinuations were toxicity (32%), treatment failure (18%), treatment simplification (5%), drug interactions (3%), and other or unspecified reasons (18%). The incidences of treatment limiting toxicity were 50.6 (95% CI 46.2–55.4), 1.6 (0.5–4.8), 2.0 (1.2–3.3), and 1.3 (0.6–2.8) per 1000 patient years for stavudine, abacavir, efavirenz and lopinavir/ritonavir respectively.

**Conclusions:**

While stavudine was associated with a high risk of treatment-limiting toxicity, abacavir, lopinavir/ritonavir and efavirenz were well-tolerated. This supports the World Health Organization recommendation to replace stavudine with abacavir or zidovudine in paediatric first-line ART regimens in order to improve paediatric first-line ART durability.

## Introduction

World Health Organization (WHO) guidelines have progressively recommended earlier antiretroviral therapy (ART) initiation for children, with the most recent WHO recommendation being immediate ART irrespective of CD4 count for all adults and children [1–4]. Children are therefore increasingly initiating ART at younger ages. Since ART must be taken lifelong and there are a limited number of paediatric antiretroviral options, knowing the long term safety and durability of ART in children is important to optimise their management. In addition, accurately forecasting future need for different antiretrovirals in children is important considering the challenges of ensuring adequate supply of appropriate pediatric antiretroviral formulations given the small and diminishing size of the pediatric epidemic relative to the adult epidemic. Knowledge of durability of currently used regimens such as abacavir and lopinavir-ritonavir is therefore vital.

In 2010 the WHO advised against the use of stavudine due to its long term toxicity, and recommended the use of either abacavir or zidovudine instead [4]. In 2013 WHO recommended lopinavir/ritonavir for first-line ART in all children less than three years old due to its superior virologic suppression [5]. The limited data on ART modifications in children from resource-limited countries suggest that fewer treatment modifications for any reason tend to occur in resource-limited settings, where the number of alternative drugs is limited [6–8]. Notwithstanding, there are very few studies describing the durability of the currently recommended abacavir- or lopinavir/ritonavir-containing first-line paediatric ART regimens in routine care [9, 10].

In South Africa, lopinavir/ritonavir has been part of recommended first-line ART for all children less than three years old since 2004, and abacavir has been the preferred nucleoside reverse transcriptase inhibitor since 2010 [11, 12]. A study in South African adults showed that the incidence of antiretroviral substitutions decreased after stavudine was replaced with tenofovir as part of the preferred first-line regimen, [13] but it is unknown whether replacing stavudine with abacavir had a similar effect on first-line ART regimen durability in paediatric cohorts.

We describe ART regimen durability and reasons for discontinuation of first-line antiretrovirals in children at two South African ART programmes from 2004–2014 covering the periods both before and after the replacement of stavudine with abacavir in first-line paediatric ART.

## Methods

### Study setting and population

We included all eligible patients from the Khayelitsha HIV Treatment Programme, which comprises three primary care clinics in Cape Town, South Africa, and from the HIV clinics at Rahima Moosa Mother and Child Hospital (RMMCH) clinic, a tertiary hospital dedicated to mother and child clinical care in Johannesburg, South Africa. Both sites provide HIV treatment services according to national guidelines and prospectively collect routine clinical data electronically. We included treatment-naïve children less than 16 years old who initiated ART with at least three antiretrovirals, between January 2004 and October 2013 (Khayelitsha) or March 2014 (RMMCH), and had at least one follow-up visit.

### Guidelines for ART initiation and treatment

South African national guideline ART eligibility and first-line regimen recommendations changed over the study period (S1 and S2 Tables) [11, 12, 14, 15]. Briefly, in 2004, ART eligibility depended on CD4 percent/count values or WHO clinical stage [11]. By 2013, ART was recommended for all children less than five years old, regardless of CD4 percent/count [14]. Before 2007, the recommended first-line ART regimens comprised stavudine and lamivudine, with efavirenz in children older than three years; lopinavir/ritonavir in those aged six months to three years; or ritonavir in those younger than six months [11, 15]. In 2007, lopinavir/ritonavir was recommended instead of ritonavir in children less than six months old [15]. From 1 April 2010, abacavir was recommended instead of stavudine, and children were electively changed to abacavir to prevent stavudine toxicity [12].

### Data management and statistical analysis

Data were captured electronically at the sites, and combined using a standard data transfer format. We looked for inconsistencies and possible errors, which were corrected by the sites to ensure completeness of ART prescription data. We used Stata 13.0 for data management and analysis.

We estimated the incidences of stavudine, abacavir, efavirenz and lopinavir/ritonavir discontinuations using Kaplan-Meier analyses. We determined the reasons for antiretroviral discontinuations using competing risks analysis. We explored associations with treatment-limiting toxicity using Cox regression. We included sex and weight-for-age z-score at ART initiation in the model *a priori*. We included age as a binary variable (<36 and ≥36 months) because of differing ART recommendations for those <36 months and ≥36 months old.

We censored patients at the first of: death, transfer out, loss to follow-up, database closure, or five years of follow-up. We considered patients lost to follow up if they had no visit for nine months before database closure and censored them at their last visit date. We did not consider treatment interruptions of less than two months to be a treatment discontinuation, unless the reason for the interruption was documented as toxicity. Treatment failure included virological, clinical, or immunological failure as defined by the treating physician using national guidelines. We defined treatment-limiting toxicity as the discontinuation of at least one antiretroviral with the reason for discontinuation documented as toxicity by the treating clinician.

Due to the 2010 guideline change that encouraged clinicians to change stavudine to abacavir to prevent toxicity, it is possible that a proportion of stavudine discontinuations after this date were actually pre-emptive to prevent toxicity rather than due to toxicity. We therefore performed a sensitivity analysis that censored patient follow-up on 01 April 2010 and excluded stavudine discontinuations that occurred after 01 April 2010.

### Ethics

Data were anonymised to ensure patient confidentiality. The University of Cape Town and University of the Witwatersrand Human Research Ethics Committees approved data collection and analysis. The study protocol was also reviewed and cleared as human subjects’ research by the U.S. Centers for Disease Control and Prevention (CDC).

## Results

### Study cohort

We included 3 579 children with median follow-up of 41 months (IQR 14.1 to 71.8) (Table 1). Four percent (142/3 579) of included children died, 75% (107/142) in the first year of ART. Nineteen percent (662/3 579) of children were lost to follow up and 18% (648/3579) were transferred to another facility. Rates of loss to follow up were similar at both sites. The proportion of children who initiated ART before 12 months of age increased from 6% in 2004 to 29% (49/170) in 2013. The most common first-line regimen was lamivudine and stavudine with either efavirenz in children older than years (61%, 1199/1954)) or lopinavir/ritonavir in children younger than three years (52%, 842/1625)).

**Table 1:** Characteristics of children initiated on antiretroviral treatment (N = 3579).

Characteristics at ART initiation		N^e^	All children N = 3579		Rahima Moosa N = 2464		Khayelitsha N = 1115	
			Median	(IQR)	Median	(IQR)	Median	(IQR)
**Age (months)**		3579	43.5	(13.4–89.2)	43.4	(12.4–91.0)	43.8	(15.2–86.1)
**CD4 percent (%)**		1993	15	(9–21)	14	(8–20)	17	(11–24)
**CD4 count (cells/mm**^**3**^**)**		2192	468	(223–852)	436	(211–791)	536	(252–980)
**Viral load (log**_**10**_ **copies)**		1840	5.3	(4.5–6.0)	5.4	(4.7–6.5)	4.9	(4.0–5.7)
**Weight-for age z score**		2379	-1.5	(-2.5 to -0.5)	-1.8	(-3.0 to -0.8)	-0.9	(-1.8 to -0.1)
			**n**	**(%)**	**n**	**(%)**	**n**	**(%)**
**Male gender**		3577	1787	(50%)	1257	(51%)	530	(48%)
**WHO clinical stage 3 or 4**		2315	1628	(70%)	865	(70%)	763	(71%)
**Severe immunosuppression**^a^		1845	1221	(66%)	947	(70%)	274	(55%)
**Severe anaemia at ART initiation**^b^		879	30	(3%)	30	(4%)	0	0
**ART regimen**^c^**:**		3579						
**NRTI**	Stavudine		2363	(66%)	1667	(68%)	696	(62%)
	Abacavir		1040	(29%)	740	(30%)	300	(27%)
	Zidovudine		161	(4%)	51	(2%)	110	(10%)
	Tenofovir		15	(0.4%)	6	(0.2%)	9	(0.8)%)
**PI or NNRTI**^d^	Efavirenz		1849	(52%)	1356	(55%)	493	(44%)
	Nevirapine		153	(4%)	42	(2%)	111	(10%)
	Lopinavir/ritonovir		1522	(43%)	1034	(42%)	488	(44%)
	Ritonavir alone		54	(2%)	31	(1%)	23	(2%)

^a^. Severe immunosuppression defined according to WHO 2006 criteria.

^b^. Severe anaemia defined as a haemoglobin of <7g/dL.

^c^. Most of the regimens were in the form NRTI+lamivudine+NNRTI/PI. Two children received emtricitabine instead of lamivudine. Percentages might not add up to 100% due to rounding off.

^d^. Unspecified in one case.

^e^. Some variables were not measured in all children

ART: antiretroviral treatment. IQR: interquartile range. NRTI: nucleoside reverse transcriptase inhibitor. NNRTI: non-nucleoside reverse transcriptase inhibitor. PI: protease inhibitor. WHO: World Health Organization

### Antiretroviral discontinuations

**Fig 1:**
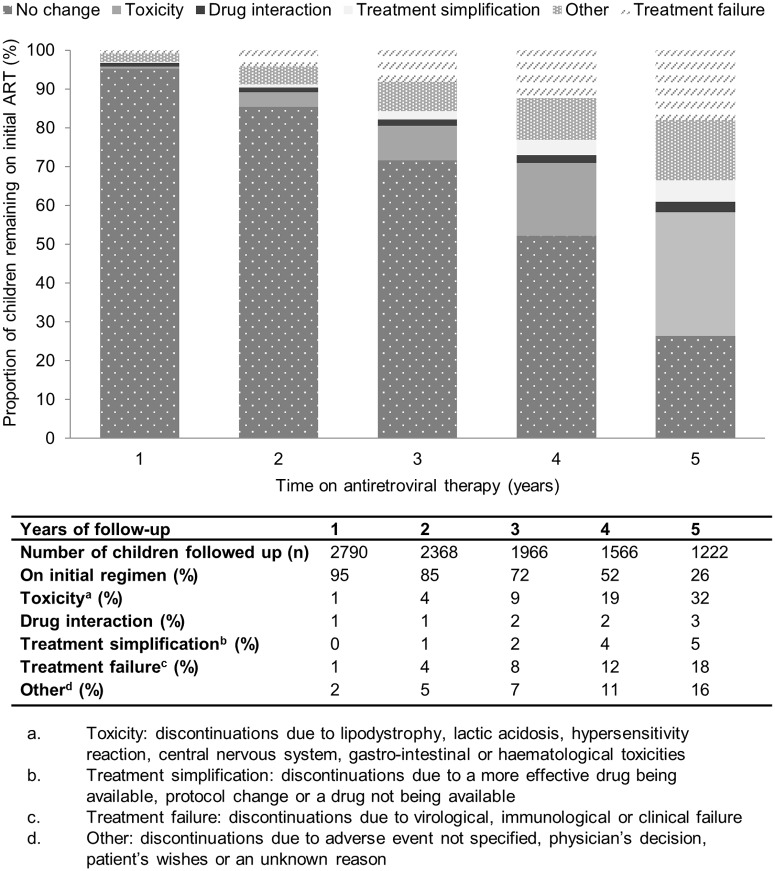
Proportion of children remaining on initial first-line antiretroviral regimen and reasons for regimen change over 5 years of follow-up.

Thirty percent (1 071/3 579) of children had an antiretroviral discontinuation. The reason for antiretroviral discontinuation was unknown in 58 (5%) children (13% at Khayelitsha and 2% at Rahima Moosa). The overall incidence of first antiretroviral discontinuation was 85.9 per 1000 patient years (py) (95% confidence interval (CI) 80.2 to 92.0). After one, three and five years 95%, 72% and 26% of children respectively remained on their initial first-line regimen (Fig 1). In the first two years on ART, the rates of discontinuations for treatment failure and for toxicity were similar, but toxicity was the most common reason for drug discontinuations from three years onwards, with a cumulative incidence of 7% and 23% at three and five years respectively. The drug most frequently discontinued was stavudine, with an incidence of 87 per 1 000 py (95% CI 81.1 to 92.8). In contrast, incidence of abacavir discontinuation was 30 per 1 000 patient years (95% CI 23.3 to 39.4). Drug-specific reasons for antiretroviral discontinuations are shown in S3 Table.

### Treatment-limiting toxicity

The overall rate of treatment-limiting toxicity was 13.4 per 1 000 py (95% CI 11.3 to 16.0). The incidence of treatment limiting toxicity was 50.6 (95% CI 46.2 to 55.4) per 1 000 patient years for stavudine, and 1.6 (95% CI 0.5 to 4.8), 2.0 (95% CI 1.2 to 3.3), and 1.3 (95% CI 0.6 to 2.8) per 1 000 patient years for abacavir, efavirenz and lopinavir/ritonavir respectively. Lipodystrophy accounted for 95% (463/483) of treatment-limiting toxicities (450 due to stavudine and seven due to efavirenz). Other causes of treatment-limiting toxicity included: hyperlactataemia in five patients on stavudine; hypersensitivity reaction in two patients on abacavir; and neurological symptoms in one patient on efavirenz.

Results from adjusted cox-regression showed that children on stavudine were 30.8 times more likely to experience treatment-limiting toxicity compared with children on abacavir (95% CI: 4.3 to 220.2). Older age (≥36 months compared with <36 months) and site were also significant predictors of treatment-limiting toxicity (Table 2).

**Table 2:** Predictors of discontinuations due to toxicity using cox-proportional hazards regression among children followed up until 5 years (n = 3579).

Variable		Unadjusted HR (95% CI)	P value	Adjusted^a^ HR (95% CI)	P value
**Site**	Khayelitsha (N = 1115)	1		1	
	Rahima Moosa (N = 2464)	2.5 (1.9 to 3.3)	<0.001	2.8 (2.0 to 3.8)	<0.001
**Age (months)**	<36 (N = 1625)	1		1	
	≥36 (N = 1954)	1.7 (1.4 to 2.2)	<0.001	1.80 (1.4 to 2.4)	<0.001
**NRTI**	Abacavir (N = 1043)	1		1	
	Stavudine (N = 2363)	17.0 (5.4 to 53.0)	<0.001	30.8 (4.3 to 220.2)	0.001
	Another NRTI (N = 1730)	0.8 (0.1 to 7.3)	0.8	4.7 (0.3 to 75.4)	0.279

^a^. Adjusted for sex and baseline weight-for-age z-score.

CI: confidence interval. HR: hazard ratio. NRTI: nucleoside reverse transcriptase inhibitor.

### Sensitivity analysis

More than two-thirds (68%, 571/841) of stavudine discontinuations occurred after 01 April 2010. In the sensitivity analysis that excluded stavudine discontinuations and follow-up after 01 April 2010, durability of first-line therapy at five years on ART increased from 26% to 52%; and the adjusted hazard ratio for treatment-limiting toxicity for children on stavudine compared with children on abacavir decreased from 30.8 to 9.9 (95% CI 1.4 to 71.8, adjusted for age, site, sex and baseline weight-for-age z-score).

## Discussion

In our cohort of 3 579 South African children, 28% and 74% of children had a first-line regimen discontinuation by three and five years on treatment respectively. The most common reason for discontinuation was toxicity (13%) followed by treatment failure (7%). Receiving stavudine and age over three years were associated with a higher risk of treatment-limiting toxicity.

Our study is one of the first to examine regimen durability and treatment-limiting toxicity after the replacement of stavudine with abacavir in first-line ART. In contrast to our findings, treatment failure was a more common reason for antiretroviral discontinuation than toxicity in other South African studies conducted before 2010. Reddi *et al*. [8] reported that 4.6% and 1.3% of discontinuations were due to treatment failure and toxicity respectively after a median follow up of eight months, and Kampiire *et al* [16] reported that 6.7% and 3.1% of discontinuations were due to treatment failure and toxicity respectively by three years on ART. This probably reflects the fact that without access to abacavir, clinicians were largely unable to switch patients off of stavudine, even if they experienced toxicity. Although our study may over-estimate stavudine toxicity as some patients were probably switched off of stavudine to prevent toxicity, rather than because of toxicity itself, it is clear that introduction of abacavir-based first-line regimens significantly reduced treatment-limiting toxicity. Only two patients experienced abacavir hypersensitivity reaction in our study. The incidence of abacavir hypersensitivity reaction in children is lower than in adults [9, 17], and African descent reduces the risk further [10, 18].

Our study shows that lopinavir/ritonavir and efavirenz were relatively well-tolerated with incidences of treatment-limiting toxicity of 1.3 (95% CI 0.6 to 2.8) and 2.0 (95% CI 1.2 to 3.3) per 1 000 patient years respectively. In contrast, studies conducted in Rwanda, Uganda and India, where were most patients were on nevirapine-based ART, reported overall probabilities of treatment-limiting toxicities of 8.3% [19], 19% [20], and 25.4% [21] respectively.

In our study, in addition to ART regimen (being on stavudine versus abacavir) older age and site were independent predictors of treatment-limiting toxicity. It is unclear why older children are more likely to experience treatment-limiting toxicity. The majority of children ≥36 months receive efavirenz while children <36 months receive lopinavir/ritonavir and, due to these age-based regimens, we could not adjust for both age and efavirenz versus lopinavir/ritonavir in the models. Consequently, we cannot exclude the possibility that efavirenz and not older age is a risk factor for a treatment-limiting toxicity. Older children are more able to verbalize when they experience toxicity compared to younger children which may be important especially for neuropsychiatric toxicity. In addition, lipodystrophy, the most common toxicity, might be easier to diagnose in older children. Similarly, only three patients reported stavudine-related peripheral neuropathy. The low incidence of peripheral neuropathy relative to adult patients is consistent with previous studies, and might be due to both lower incidence and difficulty diagnosing peripheral neuropathy in children [20, 22].

After adjusting for age, concomitant antiretrovirals, sex, and weight-for-age z-score, children at Rahima Moosa Mother and Child Hospital were 2.8 times as likely as those at Khayelitsha clinic to experience a toxicity-related drug discontinuation. Children at Rahima Moosa had lower average CD4 counts and a higher proportion of severe immune suppression than those at Khayelitsha, which might have increased their risk for drug toxicity. In addition, clinicians at Rahima Moosa, a tertiary hospital, were probably more likely to have immediate access to monitoring blood results and alternative antiretrovirals. They were also more likely to be paediatricians, who were probably more confident in recognising adverse drug reactions and switching children off of antiretrovirals than the primary care doctors and nurses at Khayelitsha clinics. The percentage of missing reasons for drug discontinuations was higher at Khayelitsha than at Rahima Moosa, so it is also possible that the effect of site on toxicity-related drug discontinuations might be overestimated.

Data were collected from routine clinic practice and we relied on the treating clinicians’ diagnoses of toxicity with likely discrepancies across sites and between clinicians. Also, our study focuses on treatment discontinuations relatively early in the course of ART as we assessed only the first discontinuation of a drug. Treatment-limiting toxicities could also have been under-estimated due to loss to follow-up, if children were lost to follow-up due to toxicity. Nevertheless, our study is a large paediatric longitudinal study with long follow-up. It spanned the periods before and after a drug protocol change occurred in South Africa, substitution of stavudine with abacavir, which enabled us to describe abacavir durability. The cohorts in this study are representative of clinic and hospital settings in South Africa and the inclusion of both settings provides a better picture of treatment-limiting toxicity than if only one setting were included. However, the generalisability to other African countries will depend on drug regimens used, availability of alternative drugs and whether clinical monitoring is comparable to our setting.

## Conclusions

Durability of first-line ART was relatively high in our study. The introduction of abacavir reduced treatment-limiting toxicity in comparison to stavudine, and the incidence of treatment-limiting toxicity for patients on lopinavir/ritonavir or efavirenz was low. Nevertheless, ongoing monitoring of the durability of first-line ART is required to accurately predict need for alternative drug options in children as infants and children start ART earlier, availability of alternative drugs and formulations increases, and children survive on ART into adolescence and adulthood.

## Supporting information

S1 Table Changes in guidelines for antiretroviral therapy initiation. (DOCX)

S2 Table Changes in the recommended first-line antiretroviral therapy. (DOCX)

S3 Table Reasons for discontinuations by antiretroviral drug. (DOCX)

## References

[1] World Health Organization. Guideline on when to start antiretroviral therapy and on pre-exposure prophylaxis for HIV. Geneva: WHO press; 2015. 26598776

[2] World Health Organization. Scaling up Antiretroviral Therapy in Resource-Limited Settings: Treatment Guidelines for a Public Health Approach, 2003 Revision. Geneva: WHO; 2004.

[3] World Health Organization. Antiretroviral therapy of HIV infection in infants and children: towards universal access Geneva: WHO; 2006. 23741772

[4] World Health Organization. Antiretroviral therapy for HIV infection in infants and children: Towards universal access Recommendations for a public health approach: 2010 revision. Geneva: WHO Press; 2010. 23741772

[5] World Health Organization. Consolidated guidelines on the use of antiretroviral drugs for treating and preventing HIV infection Recommendations for a public health approach. Geneva; WHO Press; 2013. 24716260

[6] Bracher L, Valerius NH, Rosenfeldt V, Herlin T, Fisker N, Nielsen H, et al Long-term effectiveness of highly active antiretroviral therapy (HAART) in perinatally HIV-infected children in Denmark. Scand J Infect Dis. 2007;39(9):799–804. 17701719 10.1080/00365540701203493

[7] Palladino C, Briz V, Bellon JM, Climent FJ, de Ory SJ, Mellado MJ, et al Determinants of highly active antiretroviral therapy duration in HIV-1-infected children and adolescents in Madrid, Spain, from 1996 to 2012. PLoS One. 2014;9(5):e96307 10.1371/journal.pone.0096307 24788034 PMC4006876

[8] Reddi A, Leeper SC, Grobler AC, Geddes R, France KH, Dorse GL, et al Preliminary outcomes of a paediatric highly active antiretroviral therapy cohort from KwaZulu-Natal, South Africa. BMC Pediatr. 2007;7(13):13. 17367540 10.1186/1471-2431-7-13 PMC1847430

[9] Kline MW, Blanchard S, Fletcher CV, Shenep JL, McKinney RE Jr., Brundage RC, et al A phase I study of abacavir (1592U89) alone and in combination with other antiretroviral agents in infants and children with human immunodeficiency virus infection. AIDS Clinical Trials Group 330 Team. Pediatrics. 1999;103(4):e47 10103339 10.1542/peds.103.4.e47

[10] Nahirya-Ntege P, Musiime V, Naidoo B, Bakeera-Kitaka S, Nathoo K, Munderi P, et al Low incidence of abacavir hypersensitivity reaction among African children initiating antiretroviral therapy. Pediatr Infect Dis J. 2011;30(6):535–7. 21164384 10.1097/INF.0b013e3182076864

[11] National Department of Health South Africa. National Antiretroviral Treatment Guidelines. Pretoria: Jacana; 2004.

[12] National Department of Health of South Africa. Guidelines for the Management of HIV in Children, 2nd Edition, 2010 Pretoria: NDoH; 2010.

[13] Brennan AT, Maskew M, Ive P, Shearer K, Long L, Sanne I, et al Increases in regimen durability associated with the introduction of tenofovir at a large public-sector clinic in Johannesburg, South Africa. J Int AIDS Soc. 2013;16:18794 10.7448/IAS.16.1.18794 24256692 PMC3835788

[14] South African National Department of Health. The South African Antiretroviral Treatment Guidelines 2013. Pretoria: NDoH; 2013.

[15] National Department of Health South Africa. Guidelines for the management of HIV-infected children in South Africa. Pretoria: Jacana; 2005.

[16] Kampiire L, Garone D, Giddy J, Rabie H, Wood R, Moultrie H, et al. Substitutions to initial antiretroviral therapy in children in South Africa—The IeDEA—Southern Africa Paediatric Collaboration. 19th International AIDS Conference; 2012; Washington, USA.

[17] Green H, Gibb DM, Walker AS, Pillay D, Butler K, Candeias F, et al Lamivudine/abacavir maintains virological superiority over zidovudine/lamivudine and zidovudine/abacavir beyond 5 years in children. AIDS. 2007;21(8):947–55. 10.1097/QAD.0b013e3280e087e7 17457088

[18] Hewitt RG. Abacavir hypersensitivity reaction. Clin Infect Dis. 2002;34(8):1137–42. 10.1086/339751 11915004

[19] van Griensven J, De Naeyer L, Uwera J, Asiimwe A, Gazille C, Reid T. Success with antiretroviral treatment for children in Kigali, Rwanda: experience with health center/nurse-based care. BMC Pediatr. 2008;8(39):39. 18831747 10.1186/1471-2431-8-39 PMC2570363

[20] Tukei V, Asiimwe A, Maganda A, Atugonza R, Sebuliba I, Bakeera-Kitaka S, et al Safety and Tolerability of Antiretroviral Therapy Among HIV-Infected Children And Adolescents In Uganda. J Acquir Immune Defic Syndr. 2012;59:274–80. 10.1097/QAI.0b013e3182423668 22126740

[21] Kumarasamy N, Venkatesh KK, Devaleenol B, Poongulali S, Mothi SN, Solomon S. Safety, tolerability and effectiveness of generic HAART in HIV-infected children in South India. J Trop Pediatr. 2009;55(3):155–9. 10.1093/tropej/fmn080 18829638

[22] Palmer M, Chersich M, Moultrie H, Kuhn L, Fairlie L, Meyers T. Frequency of stavudine substitution due to toxicity in children receiving antiretroviral treatment in sub-Saharan Africa. Aids. 2013;27(5):781–5. 10.1097/QAD.0b013e32835c54b8 23169331

