# Supplementary material for: First-line antiretroviral drug discontinuations in children
Source: PLoS One. 2017 Feb 13;12(2):e0169762. doi: 10.1371/journal.pone.0169762 (PMC5305232; doi:10.1371/journal.pone.0169762)
Supplement: S1 Table — (DOCX) [file pone.0169762.s001.docx]

**S1 Table: Changes in guidelines for antiretroviral therapy initiation**

| **2004** | **2010** | **2013** |
| --- | --- | --- |
| 1. Recurrent hospitalizations (>2 admissions/year) or prolonged hospitalization (>4weeks) for an HIV-related illness 2. Modified WHO stage II or III 3. <18 months: CD4% <20% or ≥18 months: CD4% <15% | 1. Children <1 year old irrespective of CD4 count 2. 1-5 years: Clinical stage 3 or 4 or CD4% ≤25% or CD4 count <750/µl 3. ≥5-15 years: Clinical stage 3 or 4 or absolute CD4 count ≤350/µl | 1. Children <5 years irrespective of CD4 count 2. ≥5-15 years: Clinical stage 3 or 4 or CD4 count ≤350/µL |
