# Supplementary material for: First-line antiretroviral drug discontinuations in children
Source: PLoS One. 2017 Feb 13;12(2):e0169762. doi: 10.1371/journal.pone.0169762 (PMC5305232; doi:10.1371/journal.pone.0169762)
Supplement: S2 Table — (DOCX) [file pone.0169762.s002.docx]

**S2 Table: Changes in the recommended first-line antiretroviral therapy**

|  | 2004-2007 | >2007 | From 01 April 2010 |
| --- | --- | --- | --- |
| <6 months | 3TC/D4T/RTV | 3TC/D4T/LPV/r | 3TC/ABC/LPV/r |
| 6 months - 3 years | 3TC/D4T/LPV/r | 3TC/D4T/LPV/r | 3TC/ABC/LPV/r |
| ≥3 years/>10kg | 3TC/D4T/EFV | 3TC/D4T/EFV | 3TC/ABC/EFV |
| Children* with TB | 3TC/D4T/RTV | 3TC/D4T/LPV/r boosted with RTV | 3TC/ABC/LPV/r boosted with RTV |

*Only in children <3years old or <10kg.

3TC-lamuvidine, D4T- stavudine, RTV-ritonavir, LPV/r-lopinavir and ritonavir, ABC-abacavir, TB-tuberculosis
