# Supplementary material for: First-line antiretroviral drug discontinuations in children
Source: PLoS One. 2017 Feb 13;12(2):e0169762. doi: 10.1371/journal.pone.0169762 (PMC5305232; doi:10.1371/journal.pone.0169762)
Supplement: S3 Table — (DOCX) [file pone.0169762.s003.docx]

**S3 Table: Reasons for discontinuations by antiretroviral drug (N=3579)**

|  | N^b^ | Toxicity^c^  n (%)^d^ | DI^e^  n (%) | | Simplification^f^  n (%) | | Failure^g^  n (%) | Other^h^  n (%) | Total  n (%) |  |
| --- | --- | --- | --- | --- | --- | --- | --- | --- | --- | --- |
| Stavudine | 2363 | 465 (93%) | 2 (5%) | | 73 (72%) | | 207 (34%) | 94 (26%) | 841 (52%) |  |
| Abacavir | 1043 | 3 (0.6%) | 0 | | 3 (3%) | | 30 (5%) | 22 (6%) | 58 (4%) |  |
| Zidovudine | 161 | 2 (0.4%) | 3 (8%) | | 0 | | 6 (1%) | 15 (4%) | 26 (2%) |  |
| Lamivudine | 3574 | 6 (1.2%) | 3 (8%) | | 10 (10%) | | 119 (20%) | 64 (18%) | 202 (13%) |  |
| Tenofovir | 15 | 0 | 0 | | 0 | | 3 (0.5%) | 1 (0.3%) | 4 (0.2%) |  |
| Efavirenz | 1849 | 15 (3%) | 1 (3%) | | 0 | | 202 (33%) | 46 (13%) | 264 (16%) |  |
| Nevirapine | 153 | 1 (0.2%) | 12 (31%) | | 0 | | 24 (4%) | 26 (7%) | 63 (4%) |  |
| Lopinavir/ritonavir | 1522 | 6 (1.2%) | 7 (18%) | | 14 (14%) | | 18 (3%) | 67 (19%) | 112 (7%) |  |
| Ritonavir | 214 | 1 (0.2%) | 11 (28%) | | 2 (2%) | | 1 (0.2%) | 23 (6%) | 38 (2%) |  |
| Total discontinuations^a^ | | 499 | | 39 | 102 | 610 | | 358 | 1608 | |
| Total patients^a^ | | 483 | | 34 | 91 | 263 | | 217 | 1071 | |

1. More than 1 drug could have been discontinued per patient
2. The number of patients initiated on the drug as first-line
3. Toxicity – discontinuations due to lipodystrophy, lactic acidosis, hypersensitivity reaction, central nervous system, gastro-intestinal or haematological toxicities
4. The column percentages (using total discontinuations as the denominator) were calculated and it might not add up to 100% due to rounding off.
5. DI – discontinuations due to drug interactions such as starting or stopping TB medications or due to comorbidities
6. Simplification – discontinuations due to a more effective drug being available, protocol change or a drug not being available
7. Failure –discontinuations due to virological, immunological or clinical failure
8. Other – discontinuations due to adverse event not specified, physician’s decision, patient’s wishes or an unknown reason
